# Clubroot resistant in cruciferous crops: recent advances in genes and QTLs identification and utilization

**DOI:** 10.1093/hr/uhaf105

**Published:** 2025-04-16

**Authors:** Shangxiang Lai, Yunshuai Huang, Yumei Liu, Fengqing Han, Mu Zhuang, Xia Cui, Zhansheng Li

**Affiliations:** State Key Laboratory of Vegetable Biobreeding, Institute of Vegetables and Flowers, Chinese Academy of Agricultural Sciences, No.12 Zhongguancun South Street, Haidian District, Beijing 100081, China; Key Laboratory of Quality and Safety Control for Subtropical Fruit and Vegetable, Ministry of Agriculture and Rural Affairs, Collaborative Innovation Center for Efficient and Green Production of Agriculture in Mountainous Areas of Zhejiang Province, College of Horticulture Science, Zhejiang A&F University, Hangzhou 311300, Zhejiang, China; Key Laboratory of Quality and Safety Control for Subtropical Fruit and Vegetable, Ministry of Agriculture and Rural Affairs, Collaborative Innovation Center for Efficient and Green Production of Agriculture in Mountainous Areas of Zhejiang Province, College of Horticulture Science, Zhejiang A&F University, Hangzhou 311300, Zhejiang, China; State Key Laboratory of Vegetable Biobreeding, Institute of Vegetables and Flowers, Chinese Academy of Agricultural Sciences, No.12 Zhongguancun South Street, Haidian District, Beijing 100081, China; State Key Laboratory of Vegetable Biobreeding, Institute of Vegetables and Flowers, Chinese Academy of Agricultural Sciences, No.12 Zhongguancun South Street, Haidian District, Beijing 100081, China; State Key Laboratory of Vegetable Biobreeding, Institute of Vegetables and Flowers, Chinese Academy of Agricultural Sciences, No.12 Zhongguancun South Street, Haidian District, Beijing 100081, China; State Key Laboratory of Vegetable Biobreeding, Institute of Vegetables and Flowers, Chinese Academy of Agricultural Sciences, No.12 Zhongguancun South Street, Haidian District, Beijing 100081, China; Key Laboratory of Quality and Safety Control for Subtropical Fruit and Vegetable, Ministry of Agriculture and Rural Affairs, Collaborative Innovation Center for Efficient and Green Production of Agriculture in Mountainous Areas of Zhejiang Province, College of Horticulture Science, Zhejiang A&F University, Hangzhou 311300, Zhejiang, China; State Key Laboratory of Vegetable Biobreeding, Institute of Vegetables and Flowers, Chinese Academy of Agricultural Sciences, No.12 Zhongguancun South Street, Haidian District, Beijing 100081, China

## Abstract

Clubroot, caused by *Plasmodiophora brassicae*, poses a serious threat to cruciferous crop production worldwide. Breeding resistant varieties remains the most cost-effective strategy to mitigate yield losses, yet achieving durable, stable, and broad-spectrum resistance continues to be a formidable challenge. Recent advances in genetic and genomic technologies have improved the understanding of complex host–pathogen interactions, leading to the identification of key resistance loci, including dominant resistance genes such as *CRa* and *Crr1*, as well as quantitative trait loci. This review discusses the genetic mechanisms governing clubroot resistance and highlights applications in breeding, such as marker-assisted selection and CRISPR/*Cas9*-based genome editing, which are accelerating the development of resistant germplasm. Furthermore, integrated management strategies, encompassing resistant cultivars, crop rotation, biocontrol agents, and soil amendments, are emphasized as critical components for sustainable disease management. This review summarizes the major resistance genes against clubroot and discusses potential strategies to address the persistent threat posed by the disease.

## Introduction

The Brassicaceae is an important plant family comprising approximately 338 genera and 3709 species [1]. This family contains numerous economically significant crops, ranging from vegetables to oil-producing species, such as radish, mustard, cabbage, broccoli, kale, cauliflower, and rape, thereby holding a significant position in global agriculture [1, 2]. However, Brassicaceae crops are subject to various disease threats, with clubroot as one of the most destructive due to its rapid spread and severe impact on yield.

Clubroot is caused by *Plasmodiophora brassicae*, an obligate soil-borne pathogen [3]. Following infection, clubroot induces the formation of swollen galls on the roots, consuming large amounts of the plant’s energy and nutrients. The swollen roots subsequently lose efficiency in nutrient and water absorption, resulting in wilting, stunted growth, and even plant death [3]. Crop yield losses in clubroot-infected fields may range from 60% to 90% [4]. Even in fields with lower pathogen densities, yield reductions of up to 60% have been observed [5]. Monoculture practices or large-scale cultivation, combined with the long-term survival of clubroot spores in the soil, further amplify the disease’s impact, presenting a substantial threat to agricultural productivity [6].

The *P. brassicae* is based on its virulence on a range of *Brassica* host plants. Over time, several pathotyping systems have been established to differentiate the various *P. brassicae* pathotypes [7–10]. Early systems, such as those by Williams [10] and Somé *et al.* [8], categorized the pathogen into different races based on their ability to infect specific *Brassica* cultivars [8–10]. More refined systems, such as the European Clubroot Differential (ECD) system and the Canadian Canola Clubroot Differential (CCD) system, have been developed to offer a more detailed classification by incorporating additional cultivars and regional variations in pathogen virulence [7–10].

**Figure 1:**
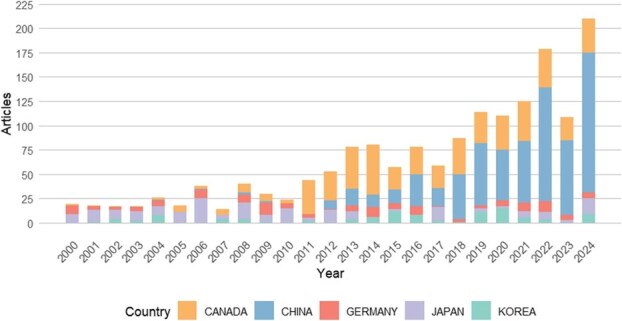
Annual publication trends on clubroot disease research from 2000 to 2024. (Data were sourced from a Scopus database search for publications on ‘clubroot’ and ‘*Plasmodiophora brassicae*’, and statistically analyzed. The literature data used in this review were retrieved on January 15, 2025.)

Since 2000, the number of publications on clubroot disease has steadily increased, with a particularly notable surge in Canada and China, driven by the economic significance of canola and various Brassicaceae vegetables, respectively [6] (Fig. 1). While clubroot disease is widespread in most Latin American countries, there remain relatively limited reports focusing on *P. brassicae* [11]. In addition, we retrieved the top 20 most active authors (1990–2024) and the top 10 most cited articles in this field (Supplementary Data Tables S1 and S2). This review summarizes recent advances in clubroot disease research in Brassicaceae, with a focus on the identification and utilization of resistance genes and quantitative trait loci (QTLs). We also discuss emerging challenges and future prospects that may guide more sustainable disease management approaches.

## Life cycle and infection characteristics

The life cycle of *P. brassicae* comprises two infection phases: primary infection of root hairs and secondary infection of cortical tissues. During the primary infection, zoospores invade root hairs, initiating pathogen colonization. In the secondary phase, the pathogen spreads into the cortical tissues, proliferating and inducing physiological changes in the host, ultimately leading to the characteristic root swelling associated with clubroot [12]. Importantly, the pathogen’s development is highly asynchronous, with different stages often coexisting within the host tissue, complicating the study of its interaction with the host [13]. For a more detailed description of its life cycle and infection mechanisms, see Kageyama and Asano [12] and Liu *et al.* [13].

*Plasmodiophora brassicae* is an obligate soil-borne pathogen, with resting spores capable of surviving in soil for prolonged periods, often exceeding 10 years [3]. Dormant spores can spread to other areas via agricultural machinery, water, animals, or wind [14]. The pathogen primarily infects root cells, proliferating and inducing the formation of characteristic swollen root galls [15, 16]. This swelling impairs water and nutrient uptake, disrupts normal physiological functions of the root, and ultimately leads to plant wilting and growth stunting. The optimal pH range for the germination of resting spores is between 5.0 and 7.0, with germination rates significantly reduced at pH values above 7.0 [17]. High moisture conditions also increase the likelihood of clubroot occurrence [3, 18]. An overview of the available diagnostic techniques for clubroot is provided in Table 1. Additionally, *P. brassicae* exhibits strong specificity in physiological pathotypes to different hosts, which increases the challenge of disease management [19].

**Table 1:** Diagnostic techniques for *P. brassicae*

Type	Technology	Application	References
PCR based	PCR	Detection of *P. brassicae* in soil or water	[20]
	qPCR	Quantification of *P. brassicae* spores	[21]; [22]
	Dot blot + qPCR	Evaluation of *P. brassicae* gene expression	[23]
	rhPCR	Identification of *P. brassicae* pathotypes	[24]
	ddPCR	Quantification of *P. brassicae* spores and distribution	[25]
	LAMP	Rapid detection of *P. brassicae*	[26]
	rhPCR + SNaPshot	Rapid differentiation of *P. brassicae* Pathotypes	[27]
Fluorescence based	Evans blue	Detection of *P. brassicae* viability	[28, 29]
	CFW-PI	Distinguishing between live and nonviable spores	[29]
Phenotypic based	HSI + CNN	Noninvasive monitoring of clubroot	[30]
	Hydroponic	Analysis of *P. brassicae* toxicity	[31]

qPCR, quantitative PCR; rhPCR, RNase H-dependent PCR; ddPCR, droplet digital PCR; LAMP, loop-mediated isothermal amplification; CFW-PI, calcofluor white-propidium iodide; HSI, hyperspectral imaging; CNN, convolutional neural networks.

## Genetic mechanisms of resistance to clubroot

The *Brassica* species described by U’s Triangle, including Chinese cabbage (*Brassica rapa*; AA, 2*n* = 20), cabbage (*Brassica oleracea*; CC, 2*n* = 18), and black mustard (*Brassica nigra*; BB, 2*n* = 16), as well as their derived allopolyploid species, oilseed rape (*Brassica napus*; AACC, 2*n* = 38), mustard (*Brassica juncea*; AABB, 2*n* = 36), and Ethiopian mustard (*Brassica carinata*; BBCC, 2*n* = 34), serve as key models for understanding genome evolution and polyploidization in plants [57].

### Diploid

**Figure 2:**
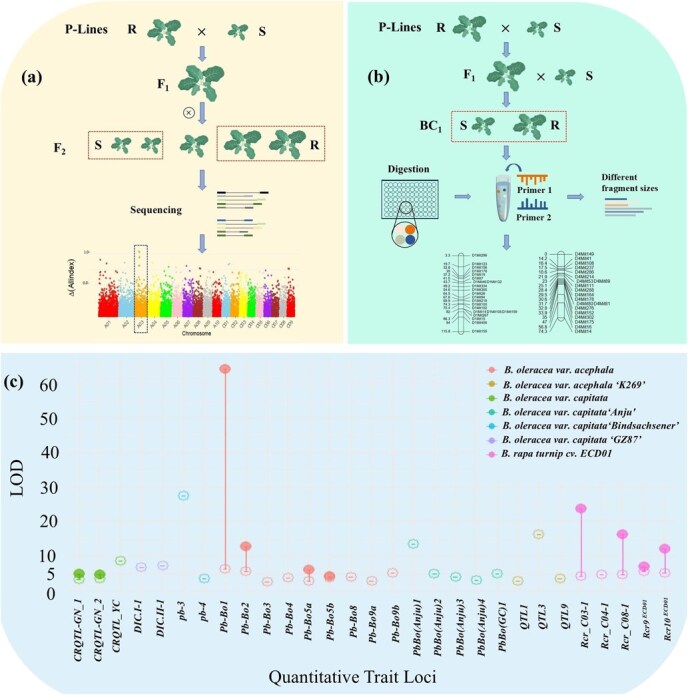
QTL mapping of CR loci in cruciferous crops. (a) BSA (bulked segregant analysis): Parental lines (P-lines), resistant (R), and susceptible (S) individuals. Extreme phenotype F_2_ individuals (red dashed boxes) from selfed F_1_ were pooled for sequencing to identify resistance-associated genomic regions and validate candidate genes. (b) GBS (genotyping-by-sequencing): The BC_1_ population, generated by backcrossing P-lines, was used as an example for GBS analysis. Genomic DNA was digested with restriction enzymes, followed by library construction and high-throughput sequencing. The resulting data were applied to genetic map construction and the identification of resistance associated genomic regions. (c) QTL analysis: LOD scores were used to assess associations between *P. brassicae* pathotypes and resistance traits. Error bars indicate the maximum and minimum LOD values observed [42, 56, 59–63].

In Chinese cabbage (*B. rapa*), clubroot resistance (CR) is primarily governed by dominant genes, with CR genes mapped using molecular markers, Genotyping-by-Sequencing (GBS), and Bulked Segregant RNA sequencing (BSA-seq) (Fig. 2). These loci are concentrated on chromosomes A03 and A08, including key genes such as *CRa*, *CRb*, *Rcr4*, and *Crr1*, which are critical for breeding resistance to multiple *P. brassicae* pathotypes (Table 2). Additional loci, such as *CrrA5* and *Crr4*, are found on chromosomes A05 and A06, while *Crr2*, *Rcr8*, and *CRc* are positioned on chromosomes A01 and A02 [32, 33, 35–38, 58].

**Table 2:** CR genes and QTLs found in *Brassica* Crops

Genes/QTLs	Chromosome	Pathogenic resistance	Source species	References
CRa	A03	P2	*B. rapa* subsp. *chinensis* line T136-8	[32]
CRb	A03	P4	*B. rapa* subsp. *pekinensis* “CR Shinki DH”	[33, 34]
CRd	A03	P4	*B. rapa* subsp. *chinensis* line 85–74	[35]
CRq	A03	P2	*B. rapa* subsp. *chinensis*	[36]
Rcr1	A03	P3	*B. rapa* subsp. *chinensis* “Flower Nabana”	[37]
Rcr2	A03	P2, P3, P5, P6, P8	*B. rapa* subsp. *chinensis* “Jazz”	[34]
CRa3.7	A03	/	*B. rapa* subsp. *pekinensis* line CR510	[38]
CRk	A03	P2, P4	*B. rapa* subsp. *rapifera* “Debra”	[39]
Crr3	A03	Ano-01	*B. rapa* subsp. *rapifera* “Milan White”	[40, 41]
Rcr10${\kern0em }^{ECD01}$	A03	3A, 3D, 3H	ECD01	[42]
BraA.CR.a	A03	/	ECD01, ECD02, ECD04	[43]
BraA3P5	A03	P5X, P5G	ECD02	[44]
X.CRa/b Kato1.1				
BraA3P5	A03	P5X, P5G	ECD02	[44]
X.CRa/b Kato1.2				
BraA.CR.c	A03	/	ECD03	[43]
PbBa3.1	A03	P2	ECD04	[45]
PbBa3.2	A03	P10	ECD04	[45]
PbBa3.3	A03	P7	ECD04	[45]
Rcr4	A03	P2, P3, P5, P6, P8	*B. rapa subsp. rapifera* “Pluto”	[46]
Rcr5	A03	P3	*B. rapa subsp. rapifera* “Purple Top White Globe”	[47]
BraA.CRb	A08	/	ECD01, ECD02, ECD03, ECD04	[43]
Rcr9${\kern0em }^{ECD01}$	A08	3A, 3D, 3H, 5X	ECD01	[42]
PbBa8.1	A08	P4	ECD04	[45]
BraPb8.3	A08	P4	*B. rapa subsp. pekinensis* line 377	[48]
CRs	A08	P4	*B. rapa subsp. chinensis* “Akimeki”	[49]
Rcr9${\kern0em }^{wa}$	A08	P5X	*B. rapa subsp. rapifera* “Pluto”, “Waaslander”	[50]
Rcr3	A08	P3H	ECD04	[50]
Rcr9	A08	P5X	*B. rapa subsp. rapifera* “Pluto”	[46]
Crr1	A08	Ano-01, Wakayama-01	*B. rapa subsp. rapifera* “Siloga”	[51]
Crr5	A08	P4	ECD01	[52]
CRc	A02	P2, P4	*B. rapa subsp. rapifera* “Debra”	[39]
Rcr8	A02	P5	*B. rapa subsp. rapifera* “Pluto”	[46]
PbBa1.1	A01	P2	ECD04	[45]
Crr2	A01	Wakayama-01	*B. rapa subsp. rapifera* “Siloga”	[51]
Crr4	A06	Ano-01, Wakayama-01	*B. rapa subsp. rapifera* “Milan White”	[53]
Rcr6	B03	P3	*B. nigra*	[54]
Rcr7	LG7	P3, P5X	*B. oleracea capitata* “Tekila”	[55]
Pb-Bo1	LG1	P1, P2, P4, P7	*B. oleracea acephala*	[56]

P, pathotypes.

Recent studies have identified two QTL, *Cr4Ba1.1* and *Cr4Ba8.1*, located on chromosomes A01 and A08, and have shown that *Bap246* confers resistance to clubroot through a recessive gene [64]. Another locus, *BraPb8.3*, was mapped to a 173.8-kb region on chromosome A08 [48]. Furthermore, resistance in *B. rapa* subsp. *rapifera* (ECD02) involves epistatic interactions between resistance genes on A03 and A08, conferring nonadditive resistance to *P. brassicae* pathotypes 3H, 5X, and 5G [44].

In *B. oleracea*, including broccoli and cabbage, resistance to clubroot is primarily controlled by QTLs [65]. Early studies identified a QTL conferring resistance to *P. brassicae* pathotype 7 in broccoli, with related QTLs later mapped in cabbage [66]. Multi-QTL mapping revealed additive effects of *pb-3* and *pb-4*, accounting for 68% of parental differences and 60% of genetic variation in double haploid (DH) lines [60]. While resistance is largely governed by additive genetic effects, dominant contributions are not entirely excluded [67]. Key loci, such as *PbBo1*, provide broad-spectrum resistance to multiple pathotypes, contributing between 20.7% and 80.7% to phenotypic variation [56]. Recent GBS efforts have identified two major QTLs, *Rcr_C03-1* and *Rcr_C08-1*, on chromosomes C03 and C08, along with five minor QTLs on chromosomes C01, C03, C04, and C08 [59]. Notably, resistance loci exhibit distinct patterns between the A and C genomes, highlighting the genetic divergence within the *Brassica* genus.

In black mustard (*B. nigra*), although studies on resistance are relatively limited, a resistance locus, *Rcr6*, located on chromosome B3, has been identified, exhibiting resistance to multiple strains [54].

### Tetraploid

*Brassica napus* (rapeseed) is an allotetraploid species, derived from *B. rapa* and *B. oleracea*, integrating the A and C genomes. Approximately 20 QTLs and resistance loci associated with clubroot resistance have been identified in *B. napus* [68]. The resistance locus *Pb-Bn1*, located on linkage group (LG) DY4, confers resistance to the *PB137-522* strain [69]. Metabolomic analyses have indicated that resistance to *P. brassicae* infection in *B. napus* is associated with multiple QTLs, each linked to distinct metabolic modules, suggesting the involvement of diverse cellular mechanisms [70]. Linkage analysis identified three QTLs: *PbBn_di_A02*, *PbBn_di_A04*, and *PbBn_di_C03* [71]. Additionally, genome-wide association studies (GWAS) have identified two QTLs on chromosomes A03 and A08, which are associated with resistance to pathotypes 3H, 3A, and 3D [72]. Studies on *B. napus* ssp. *napobrassica* have identified a major resistance locus on chromosome A08 that confers resistance to multiple *P. brassicae* pathotypes [73]. Additionally, GWAS have shown that resistance-related loci are concentrated in the upper and lower segments of chromosome A03 and the central segment of chromosome A08 [74].

*Brassica juncea* (Indian mustard) is an allotetraploid species that originated from natural hybridization between *B. rapa* and *B. nigra*. Compared to *B. napus*, fewer genetic studies have focused on *B. juncea* [75]. To introduce clubroot resistance into *B. juncea*, resynthesized *B. juncea* lines were developed through interspecific crosses between resistant *B. rapa* and susceptible *B. nigra* lines. These resynthesized lines exhibited resistance to *P. brassicae* pathotype 3 in early generations [76]. However, partial loss of resistance was observed in some self-pollinated progenies, indicating potential instability in resistance inheritance [76].

Studies have shown that during the production of DH lines in *B. napus*, the CR gene from the donor parent (ECD04) may be lost, possibly due to genetic recombination or selection pressures [77]. Although numerous CR genes have been widely identified in various cruciferous crops [35, 61], their precise genetic mechanisms remain largely unclear [32, 65]. In general, clubroot resistance is controlled by multiple loci, including both major genes and QTL, some of which function in a recessive manner [64, 78]. For example, in *B. napus*, a model based on four resistance genes has been proposed, with resistance resulting from the combined action of both dominant and recessive genes located on different chromosomes [79, 80]. Additionally, the progeny of self-pollinated resynthesized *B. napus* often exhibit distinct resistance profiles compared to hybrid progeny, likely influenced by gene segregation and epistatic effects [78]. Despite variations in genetic backgrounds and CR mechanisms across *Brassica* species, most known resistance genes display pathotype-specific or race-specific efficacy, meaning their effectiveness is restricted to particular *P. brassicae* populations [3, 45, 65].

## Clubroot resistance genes

The rapid advancement of next-generation sequencing technologies, along with decreasing costs, has made whole-genome sequencing increasingly accessible. *Brassica rapa*, the first reference genome within the *Brassica* genus has paved the way for subsequent reference genomes, including *B. napus* [81], *B. rapa* Z1 [82], *B. oleracea* HDEM [82], *B. oleracea* L. var. *botrytis* [83], *B. rapa* [84], and *B. juncea* [84]. These genomic resources have been instrumental in identifying CR genes and developing molecular markers, thereby advancing clubroot resistance research [85, 86].

### Clubroot resistance

The plant immune system defends against a wide range of pathogens throughout the plant’s lifecycle [87]. This system primarily consists of Pattern-Triggered Immunity (PTI) and Effector-Triggered Immunity (ETI) [88]. PTI is activated by the recognition of pathogen-associated molecular patterns, which trigger a basal defense response to restrict pathogen invasion [89, 90]. However, some pathogens secrete effector proteins that suppress PTI; in response, plants recognize these effectors through *R* proteins, which activate the ETI pathway, leading to a specific defense against pathogens [91]. Most effector receptors (ETRs) are encoded by resistance genes (R genes), which typically contain nucleotide-binding site (NBS) and leucine-rich repeat (LRR) domains. These domains enable plants to recognize and respond to pathogens through a specialized immune defense system [32, 89, 90].

CR genes can be broadly categorized into major-effect genes and QTL regions, with the latter often representing genomic regions where causal genes have yet to be precisely identified [65]. When resistant and susceptible phenotypes are clearly distinguishable, it becomes more feasible to perform precise mapping and gene cloning (Fig. 2a, b). Major effect R genes are relatively straightforward to identify, as they often exhibit clear phenotypic differences between resistant and susceptible plants and follow Mendelian inheritance patterns, which simplifies the process of resistance breeding and gene cloning [92]. However, the analysis of QTLs poses greater challenges because these loci often span hundreds of candidate genes, many of which may not directly contribute to resistance [93].

Recent advancements in genomic technologies, such as high-throughput sequencing and fine-mapping techniques, have created opportunities to overcome these challenges [94]. By integrating functional genomics with classical positional cloning strategies, researchers can more accurately dissect QTLs and identify minor genes that play critical roles in quantitative resistance [93, 95].

### CRs

CR genes play a critical role in conferring resistance to *P. brassicae* and are primarily located on the A genome of *Brassica* species. The first identified CR locus, *CRa*, was mapped to chromosome A3 [32]. While many CR genes have been mapped through QTL analysis, most remain uncloned.

Many CR genes are thought to originate from conserved major resistance clusters (MRCs) in ancestral *Brassica* genomes, as evidenced by their high sequence homology across species [53, 58]. Comparative genomic analyses suggest that *Brassica* species evolved from a paleohexaploid ancestor [96]. Microsynteny analysis reveals that *B. rapa* has retained the impact of three rounds of whole-genome duplication relative to *Arabidopsis thaliana*, leading to the retention of numerous functional genes despite distinct genome fractionation patterns [86]. For example, *pb-Bo(Anju)1* and *pb-Bo(Anju)2* on *B. oleracea* chromosome C02 exhibit collinearity with the *CRc* region on *B. rapa* chromosome A02 [62, 97]. Similarly, phylogenetic and synteny analyses suggest that resistance loci such as *CRA3.7.1* and *CRA8.2.4* originated from a common ancestral gene before the *Brassica* genome triplication [58]. These findings indicate that whole-genome duplications have played a key role in the diversification and retention of CR genes in *Brassica* species, alongside other evolutionary mechanisms.

Comparative mapping revealed that CR genes such as *Crr2*, *CRc*, *Crr4*, and *Crr1* in *B. rapa* are located in syntenic regions of *B. oleracea*, suggesting partial conservation of genomic structure between these species [62]. Similarly, *CRb* and *Crr3* in the R3 linkage group of *B. rapa* show analogous relationships [33, 62]. In addition, *B. napus CRa* and *Rcr1* have homologous regions on BnA03 and BnC07, corresponding to their counterparts in *B. rapa* and *B. oleracea* [93]. Notably, a region on *B. oleracea* chromosome C7 corresponds to a disease-resistance region on *B. rapa* chromosome A3, both encoding TIR-NBS-LRR proteins [55]. These conserved syntenic relationships highlight the common ancestral origin and diversification of resistance loci across the *Brassica* genus.

### Crrs

The *Crr* genes represent another extensively studied group of clubroot resistance genes, originally cloned from Chinese cabbage and cabbage. The *Crr1*, *Crr2*, *Crr3*, *Crr4*, and *Crr5* genes are located on different chromosomes and are likely associated with resistance to specific strains of *P. brassicae* [40, 51, 53]. *Crr1*, located on chromosome A08 in Chinese cabbage, is composed of two genes, *Crr1a* and *Crr1b* [51]. *Crr1a* encodes a TIR-NBS-LRR-type resistance protein that is expressed in the stele and root tissues [51, 98]. Additionally, analysis of the DH40 line, derived from a hybrid between European turnip (ECD01) and two Chinese cabbage varieties, revealed that Crr5 is also located on chromosome A08 and encodes a nucleotide-binding leucine-rich repeat (NLR) protein [52]. *Crr3*, located on chromosome A03, encodes an NBS-LRR protein that specifically recognizes the Ano-01 isolate [40, 51, 53]. *Crr4*, located on chromosome A06, confers resistance to two *P. brassicae* isolates [53]. Additionally, homology analysis suggests that *Crr1* and *Crr2* share an overlapping region with a segment on chromosome 4 of *A. thaliana*, a common origin from a shared ancestral genome [51, 53].

### Pbs

*PbBa8.1* is a clubroot resistance gene identified in *B. napus*, located on chromosome A08, and confers resistance to multiple *P. brassicae* strains, particularly pathotype 4, which is widespread in China’s major rapeseed production areas [45]. Marker-assisted backcross breeding (MABB) has been used to introgress *PbBa8.1* into elite breeding lines. Specifically, *PbBa8.1* was introduced into the Ogura CMS restorer line R2163, leading to the development of R2163R, a clubroot-resistant restorer line [99]. Molecular markers linked to *PbBa8.1*, including kompetitive allele-specific PCR (KASP) markers, have been developed and implemented in breeding programs to enhance selection efficiency [100, 101]. Other *Pb* loci have been identified but remain underutilized, requiring further research.

### Other QTLs

Clubroot resistance is primarily polygenic, with approximately 50 resistance QTLs identified across various populations and subspecies, including those found in broccoli, kale, and cabbage [102]. Differences in logarithm of odds (LOD) scores indicate varying degrees of association between clubroot resistance and specific loci across different *Brassica* subspecies, as detected in QTL analysis (Fig. 2c). Additionally, genotyping technologies, such as GBS and SNP arrays, have significantly accelerated the pace of QTL identification [103, 104]. However, due to marker discrepancies and the diversity of resistance sources across studies, direct comparison and integration of these QTLs remain challenging [105]. Notably, resistance levels in progeny did not exceed those of the parents, suggesting that most QTLs do not contribute to clubroot resistance, and resistance/susceptibility QTLs tend to converge toward the parental genotypes [62].

## Creation and utilization of resistance resources

### Identification of resistance resources

Screening for clubroot-resistant germplasm is crucial for establishing a robust genetic foundation in breeding programs. Crisp *et al.* [106] evaluated approximately 1000 cabbage (*B. oleracea*) varieties and found reduced susceptibility in Brussels sprouts, cabbage, cauliflower, and broccoli. Additionally, Peng *et al.* [107] screened 955 *Brassica* species and identified 35 varieties (primarily Chinese cabbage) with a disease severity reduction of at least 50% following inoculation with clubroot pathotype 3, among which 15 varieties exhibited complete resistance. Hasan *et al.* [108] assessed 275 *Brassica* varieties for resistance to five Canadian *P. brassicae* strains and identified several varieties resistant to multiple strains, with the majority of resistant varieties originating from species with the A genome. Xie *et al.* [109] identified and developed 41 highly resistant Brassicaceae varieties, including broccoli and other *Brassica* materials, between 2020 and 2021. Notably, 90% of these materials carried more than two clubroot resistance genes, with some possessing as many as seven genes. Ma *et al.* [110] analyzed clubroot-resistant resources in 268 radish varieties from multiple countries and identified six germplasm resources exhibiting high resistance. In addition to these efforts, private breeding programs have also contributed valuable resistant materials and germplasm [111]. Together, these findings highlight the importance of systematic germplasm screening identify novel resistance sources.

### Conventional breeding and marker-assisted selection

Genetic diversity across species or genera provides valuable resistance resources for crop improvement. Resistance genes from wild relatives or distantly related species can be introduced into target crops through hybridization and backcrossing, though genetic compatibility and reproductive barriers often limit their success [112]. Initially, researchers employed artificial hybridization and natural pollination, using methods such as grafting, mixed pollination, and chemical treatments to improve hybridization efficiency; however, these methods exhibited low success rates [105]. This limitation may be attributed to genetic differences between parents, such as chromosome number and physiological characteristics. With the advent of embryo rescue techniques, the *in vitro* culture of immature embryos has reproductive barriers in interspecific hybridization [105, 113]. For example, Liu *et al.* [114] successfully transferred CR genes from Chinese cabbage to *B. napus* through distant hybridization and embryo rescue, for the development of clubroot-resistant breeding materials.

Conventional breeding remains the primary method for utilizing resistance resources, introducing resistance genes into target crop varieties through hybridization and backcrossing. The application of molecular marker-assisted selection (MAS) has improved the efficiency of resistance breeding by facilitating the effective tracking and screening of disease resistance genes during the breeding process.

The Ogura CMS system is widely utilized in hybrid breeding of *Brassica* vegetables; however, the absence of fertility restorer genes restricts its use in hybrid breeding programs, posing challenges for the stable incorporation of resistance genes. Various breeding strategies, including cytoplasmic replacement, microspore culture, and MAS, have been employed to overcome these limitations and successfully introduce clubroot resistance genes into hybrid lines. Ren *et al.* [115] successfully transferred the clubroot resistance gene *CRb* from Ogura CMS cytoplasm into progeny with normal cytoplasm and resistance loci through MAS and the novel recovery material 16Q2-11. By cytoplasmic replacement and microspore culture, researchers overcame the sterility issues associated with Ogura CMS, cultivating *CRa*-positive microspore-derived plants [112]. Using MABB, the Ogura CMS restorer line R2163 was successfully improved by introducing the resistance locus *PbBa8.1* into *B. napus* [99]. Similarly, hybridization resistant mustard subspecies has produced S1 and S2 mustard lines with resistance to specific clubroot pathotypes [76].

However, caution is required, as undesirable traits may be closely linked with the target traits within the genome. For instance, Zhan *et al.* [101] unintentionally introduced the adjacent *Fatty Acid Elongase 1 (FAE1)* gene while breeding clubroot-resistant *B. napus*, resulting in increased erucic acid content in seeds. Notably, while undesirable genes can be removed through backcrossing and MAS, the breeding process may require additional time [116].

### Gene editing technology

The rapid development of gene-editing technologies has greatly facilitated the of resistance resources. Techniques such as CRISPR/*Cas9* enable precise editing of genes related to clubroot resistance, allowing for targeted modifications that influence plant immunity [117]. For instance, the deletion of the clubroot resistance gene *RPB1* renders two *A. thaliana* ecotypes susceptible to *P. brassicae* pathotype P1+, knocking out *miR395-APS4* enhances clubroot resistance in *B. napus* [117, 118]. In a recent study, an improved CRISPR/*Cas9* system was employed to generate clubroot-resistant *B. napus* germplasm carrying the *Rcr1* marker, without the need for selectable markers, in just 2 years [119]. Additionally, gene editing can be used to modify the promoter regions of resistance genes, thereby increasing their expression and enabling plants to respond more sensitively and rapidly to pathogen infection [120, 121].

Despite its transformative potential, challenges such as off-target effects, variable editing efficiencies across species, and regulatory constraints limit its adoption. Integrating CRISPR with traditional breeding methods, such as MAS, could optimize the development of durable resistance [122].

### Epigenetic regulation

Epigenetics refers to heritable changes in gene expression that do not alter the DNA sequence, mediated by mechanisms such as DNA methylation, noncoding RNAs, histone modifications, and chromatin remodeling [123]. For example, in *B. napus*, 24 lncRNAs on chromosome A08 responded to *P. brassicae* infection, with only eight expressed in resistant plants, suggesting their involvement in regulating defense pathways [124, 125]. In *A. thaliana*, epigenetic recombinant inbred lines identified 20 epigenetic QTLs (*QTL^sepi^*) associated with root rot resistance, with 16 directly linked to DNA methylation changes [126]. Some of these loci colocalize with resistance genes, indicating an interplay between genetic and epigenetic regulation. Similarly, DNA methylation was found to regulate the function of two adjacent NLR genes (*AT5G47260* and *AT5G47280*), highlighting its involvement in clubroot resistance [127].

However, DNA methylation can be partially or completely reset during plant reproduction [123, 128, 129]. Additionally, the activation of transposable elements, typically silenced by DNA methylation, may lead to genomic instability and interfere with the long-term inheritance of resistance traits [123, 129]. Despite these challenges, epigenetic mechanisms offer new avenues for innovative breeding strategies to enhance plant disease resistance.

### Clustering of CR genes and QTLs

Gene pyramiding has demonstrated advantages in breeding resistant cultivars, offering more durable and broad-spectrum protection against evolving pathogens. As early as 1952, Watson and Singh pioneered the pyramiding of rust resistance genes in wheat [130]. In the context of clubroot, single-gene resistant cultivars often lose effectiveness after prolonged cultivation (4–5 years), as they target only specific pathotypes [9, 111]. Cultivars harboring multiple CR loci have similarly shown enhanced resistance [131]. Moreover, relying solely on a single dominant CR gene or the accumulation of minor-effect CR-QTLs is typically insufficient for achieving durable resistance [132]. In contrast, combining a major CR gene with two or three minor genes can provide moderate yet more stable resistance, offering partial protection against multiple pathogen strains [132]. Additionally, cultivars stacking multiple CR genes exhibit stronger resistance in soils infested with *P. brassicae* [133, 134].

Recent transcriptomic data further support the efficacy of gene stacking: Wen *et al.* [133, 134] reported that lines carrying multiple resistance genes exhibited higher transcription levels of key plant immunity-related DEGs compared to lines with a single CR gene, leading to enhanced clubroot resistance in canola [133, 134]. Applying MAS, Matsumoto *et al.* [135] successfully pyramided *CRa*, *CRk*, and *CRc* in *B. rapa*, thereby conferring high resistance against multiple pathogen strains [135]. Notably, stacking CR genes also helps reduce the accumulation of pathogen in the soil [132, 136].

Similarly, transferring three CR genes from *B. rapa* into the cabbage genome significantly enhanced cabbage’s resistance to clubroot [112]. Meanwhile, Song *et al.* [137] showed that accumulating multiple CR loci in newly bred canola lines leads to broad-spectrum resistance against diverse *P. brassicae* isolates [137]. Gene pyramiding has also proven effective in other gene combinations, such as *CRb* with *PbBa8.1* [138] or *CRa* with *CRd* [139], both substantially improving resistance against multiple pathotypes. Recent studies have reported that the *WeiTsing* (WTS) gene from *Arabidopsis* confers broad-spectrum resistance to multiple clubroot isolates and exhibits the same resistance in transgenic canola, demonstrating potential for application [140].

### Deployment of CR genes

In recent years, CR varieties have become a key strategy for managing clubroot disease. The first CR canola variety in Canada, *‘45H29’*, was launched in 2009 [9]. Similarly, the winter canola variety ‘*Mendel*’, resistant to clubroot, was commercialized in Europe [141], followed by the release of several other resistant varieties. In China, successful development and deployment of resistant varieties such as ‘*Hua You Za 62R*’, ‘*Hua Shuang 5*’, ‘*Zhong Shuang 11R*’, and ‘*W3R*’ have provided valuable means of controlling specific *P. brassicae* strains [99, 142].

The effective use of resistance genes can enhance crop disease resistance. For instance, *CRb* and *PbBa8.1* confer robust resistance against *P. brassicae* pathotype 4—dominant in many regions of China—and cultivars incorporating these genes have consistently shown high levels [138]. However, maintaining the long-term effectiveness of resistant varieties is challenging. Pathogen diversification frequently results in the emergence of new virulent phenotypes [143]. For instance, continuous cultivation of resistant varieties in infected soils may contribute to the rapid proliferation of *P. brassicae* pathotype 5 [144]. Additionally, the genetic foundation of many resistant varieties is often proprietary, limiting effective resistance management strategies, particularly for rotating resistant varieties in infected fields [145].

The deployment of resistant varieties with temporal and spatial diversity is a feasible strategy for improving the management of clubroot [146–150]. Temporal diversity can be achieved through practices such as crop rotation, alternating resistance genes, or planting cover crops [151–153]. Spatial diversity can be promoted through variety mixtures or intercropping, which increases field-level species diversity and reduces the prevalence of virulent pathogen strains (see review by Botero-Ramirez *et al.* [154]).

Modern breeding programs often reuse elite parental lines to accelerate the development of high-performing cultivars [92]. This approach has significantly improved crop yield and agronomic traits, yet its effects on genetic diversity vary across crops and breeding systems [103, 155, 156]. Nonelite germplasm can serve as a valuable reservoir of resistance genes, but its direct use in breeding is often constrained by linkage drag and other agronomic disadvantages, making it less practical in certain programs [101]. Nevertheless, breeding programs commonly employ strategies such as germplasm introgression, hybridization, and MAS to integrate desirable traits while maintaining genetic diversity.

The formation of polygenic resistance involves the combination of multiple loci, each contributing small individual effects. For example, in a study aimed at achieving broad-spectrum resistance to wheat stripe rust, five R genes were incorporated into a single vector and introduced into wheat [157]. The resulting lines containing these five genes exhibited broad-spectrum resistance to diverse stripe rust pathotypes [157]. This approach demonstrates the potential of polygenic resistance but is inherently more challenging than transferring single-gene resistance due to the complexity of gene stacking and expression regulation [133, 134, 136].

Recurrent selection has proven to be an effective method for developing polygenic resistance [112, 135]. This cyclical process of recombination and selection among complementary parent lines enriches favorable alleles for disease resistance [158]. Moreover, this strategy addresses the limitations of single-gene resistance and aligns with breeding objectives for improving traits with low heritability, such as disease resistance, extended plant longevity, and enhanced overall crop performance [158–160].

## Perspective

Clubroot poses a significant threat to cruciferous crop production, with most recognized resistance loci traced to the A genome of Chinese cabbage [58]. Currently, research on clubroot primarily focuses on identifying novel CR genes and incorporating them into target crops to achieve broader and more durable resistance. The widespread application of molecular markers has facilitated the aggregation of resistance genes through MAS. However, over time, the continuous emergence of new virulent *P. brassicae* strains poses a risk of resistance breakdown [111]. In Canadian surveys, the increasing diversity of *P. brassicae* pathotypes has been highlighted. For example, pathogen population analyses from 166 fields between 2017 and 2018 identified 17 pathotypes on the CCD set, including previously unreported types such as 2C, 6D, and 13B [161]. To sustain long-term and broad-spectrum resistance, future research should prioritize an in-depth analysis of pathogen infection mechanisms and explore alternative strategies alongside the traditional deployment of resistance genes [162].

Recent advances in genomics, including GWAS and whole-genome resequencing, in combination with MAS, genomic selection (GS), and genome editing technologies (e.g. CRISPR/*Cas9*), have accelerated the identification and utilization of resistance genes [163]. Expanding the gene pool through wild relatives and underutilized germplasm remains a promising strategy [104, 164–166]. For example, the causal gene *BoUGT76C2* from wild *B. oleracea* confers clubroot resistance [166]. Further elucidation of *P. brassicae* virulence factors—via multi-omics analyses and effector characterization—may also pave the way for novel resistance strategies.

**Figure 3:**
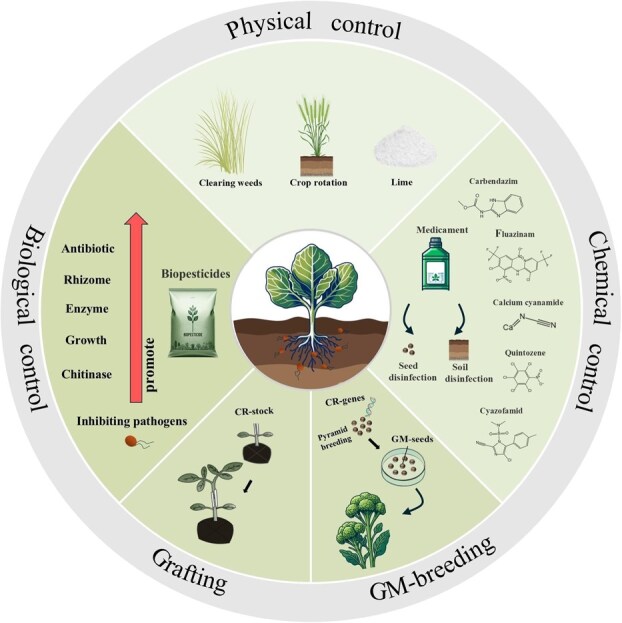
Integrated strategies for clubroot control. GM-breeding: Genetically modified breeding. CR: Clubroot resistance. CR-stock: Clubroot-resistant rootstocks. A crop is grafted onto a CR-stock to confer resistance to *P. brassicae* (created with BioRender.com).

Sustainable management strategies complement genetic approaches by leveraging soil microbiome manipulation (e.g. promoting beneficial microbial activity), deploying biocontrol agents, and optimizing agronomic practices (e.g. crop rotation and soil pH adjustment) [147, 149, 167–169] (Fig. 3). Moreover, emerging technologies such as nanotechnology and biostimulants (including microbial agents like *Trichoderma* and *Bacillus*, as well as nonmicrobial agents like seaweed extracts or melatonin) have shown promise for enhancing disease resistance with minimal chemical inputs [170–173]. Specifically, combining melatonin (MT) and copper oxide nanoparticles (CuO-NPs) was found to significantly lower clubroot severity in *B. rapa* by boosting antioxidant defenses and hormone signaling [174]. Likewise, magnesium oxide nanoparticles (MgO NPs) can promote soil microbial diversity and stimulate plant immune responses [171]. However, research on such nanotechnology-based interventions is still in its early stages.

New high-value cruciferous crops may be developed and improved, such as broccoli or broccolini crossed with *Isatis tinctoria* L. (Brassicaceae). So, it would be a strategy to achieve immune pathways through grafting with cucurbitaceous rootstocks. Consequently, integrating multiple resistance genes and adopting comprehensive management strategies can improve the prevention and clubroot in cruciferous crops.

## Supplementary Material

Web_Material_uhaf105

## References

[1] Warwick S . Brassicaceae in agriculture. In: Schmidt R, Bancroft I, eds. Genetics and Genomics of the Brassicaceae. Plants Genetics and Genomics: Crops and Models Series, Vol. 9. New York: Springer, 2011, 33–65

[2] Huang J, Liu Y, Han F. et al. Genetic diversity and population structure analysis of 161 broccoli cultivars based on SNP markers. Hortic Plant J. 2021;7:423–33

[3] Dixon GR . The occurrence and economic impact of *Plasmodiophora brassicae* and clubroot disease. J Plant Growth Regul. 2009;28:194–202

[4] Pageau, D, Lajeunesse, J, and Lafond, J (2006), 'Impact of clubroot [Plasmodiophora brassicae] on the yield and quality of canola', Canadian Journal of Plant Pathology, 28 (1), 137–43.

[5] Strehlow B, de Mol F, Struck C. Risk potential of clubroot disease on winter oilseed rape. Plant Dis. 2015;99:667–75 30699684 10.1094/PDIS-05-14-0482-RE

[6] Askarian H, Akhavan A, González LG. et al. Genetic structure of *Plasmodiophora brassicae* populations virulent on clubroot resistant canola (*Brassica napus*). Plant Dis. 2021;105:3694–704 33507096 10.1094/PDIS-09-20-1980-RE

[7] Kuginuki Y, Yoshikawa H, Hirai M. Variation in virulence of *Plasmodiophora brassicae* in Japan tested with clubroot-resistant cultivars of Chinese cabbage (*Brassica rapa* L. ssp. *pekinensis*). Eur J Plant Pathol. 1999;105:327–32

[8] Somé A. et al. Variation for virulence on *Brassica napus* L. amongst *Plasmodiophora brassicae* collections from France and derived single-spore isolates. Plant Pathol. 1996;45:432–9

[9] Strelkov SE, Hwang S-F, Manolii VP. et al. Virulence and pathotype classification of *Plasmodiophora brassicae* populations collected from clubroot resistant canola (*Brassica napus*) in Canada. Can J Plant Pathol. 2018;40:284–98

[10] Williams PH . A system for the determination of races of *Plasmodiophora brassicae* that infect Cabbage and Rutabaga. Phytopathology. 1996;56:624–26

[11] Botero A, García C, Gossen BD. et al. Clubroot disease in Latin America: distribution and management strategies. Plant Pathol. 2019;68:827–33

[12] Kageyama K, Asano T. Life cycle of *Plasmodiophora brassicae*. J Plant Growth Regul. 2009;28:203–11

[13] Liu L, Qin L, Zhou Z. et al. Refining the life cycle of *Plasmodiophora brassicae*. Phytopathology. 2020;110:1704–12 32407251 10.1094/PHYTO-02-20-0029-R

[14] Botero-Ramirez A, Hwang S-F, Strelkov SE. *Plasmodiophora brassicae* inoculum density and spatial patterns at the field level and relation to soil characteristics. Pathogens. 2021;10:499 33919064 10.3390/pathogens10050499 PMC8143121

[15] Murakami H, Tsushima S, Akimoto T. et al. Quantitative studies on the relationship between plowing into soil of clubbed roots of preceding crops caused by *Plasmodiophora brassicae* and disease severity in succeeding crops. Soil Sci Plant Nutr. 2004;50:1307–11

[16] Rajbanshi S, Pokharel A. Unveiling the clubroot pathogen *Plasmodiophora brassicae*: insights into its biology, pathogenicity, and control strategies. Russ J Agric Socioecon Sci. 2023;139:159–71

[17] Rashid A, Ahmed HU, Xiao Q. et al. Effects of root exudates and pH on *Plasmodiophora brassicae* resting spore germination and infection of canola (*Brassica napus* L.) root hairs. Crop Prot. 2013;48:16–23

[18] Gossen BD, Adhikari KKC, McDonald MR. Effects of temperature on infection and subsequent development of clubroot under controlled conditions. Plant Pathol. 2012;61:593–9

[19] Feng J, Xiao Q, Hwang S-F. et al. Infection of canola by secondary zoospores of *Plasmodiophora brassicae* produced on a nonhost. Eur J Plant Pathol. 2012;132:309–15

[20] Faggian R, Bulman SR, Lawrie AC. et al. Specific polymerase chain reaction primers for the detection of *Plasmodiophora brassicae* in soil and water. Phytopathology. 1999;89:392–7 18944752 10.1094/PHYTO.1999.89.5.392

[21] Agarwal A, Kaul V, Faggian R. et al. Analysis of global host gene expression during the primary phase of the *Arabidopsis* thaliana–*Plasmodiophora brassicae* interaction. Funct Plant Biol. 2011;38:462–78 32480901 10.1071/FP11026

[22] Faggian R, Strelkov SE. Detection and measurement of *Plasmodiophora brassicae*. J Plant Growth Regul. 2009;28:282–8

[23] Feng J, Hwang S-F, Strelkov SE. Assessment of gene expression profiles in primary and secondary zoospores of *Plasmodiophora brassicae* by dot blot and real-time PCR. Microbiol Res. 2013;168:518–24 23523193 10.1016/j.micres.2013.02.011

[24] Yang Y, Zuzak K, Harding M. et al. DNA sequence dimorphisms in populations of the clubroot pathogen *Plasmodiophora brassicae*. Plant Dis. 2018;102:1703–7 30125173 10.1094/PDIS-02-18-0225-RE

[25] Wen R, Lee J, Chu M. et al. Quantification of *Plasmodiophora brassicae* resting spores in soils using droplet digital PCR (ddPCR). Plant Dis. 2020;104:1188–94 32065569 10.1094/PDIS-03-19-0584-RE

[26] Gossen BD, al-Daoud F, Dumonceaux T. et al. Comparison of techniques for estimation of resting spores of *Plasmodiophora brassicae* in soil. Plant Pathol. 2019;68:954–61

[27] Tso HH, Galindo-González L, Locke T. et al. Protocol: rhPCR and SNaPshot assays to distinguish *Plasmodiophora brassicae* pathotype clusters. Plant Methods. 2022;18:91 35780127 10.1186/s13007-022-00923-w PMC9250251

[28] Harding MW, Hill TB, Yang Y. et al. An improved Evans blue staining method for consistent, accurate assessment of *Plasmodiophora brassicae* resting spore viability. Plant Dis. 2019;103:2330–6 31298992 10.1094/PDIS-05-18-0855-RE

[29] Wang Y, Koopmann B, von Tiedemann A. Methods for assessment of viability and germination of *Plasmodiophora brassicae* resting spores. Front Microbiol. 2021;12:823051 35069518 10.3389/fmicb.2021.823051 PMC8767001

[30] Feng L, Wu B, Chen S. et al. Application of visible/near-infrared hyperspectral imaging with convolutional neural networks to phenotype aboveground parts to detect cabbage *Plasmodiophora brassicae* (clubroot). Infrared Phys Technol. 2022;121:104040

[31] Salih R, Brochu A-S, Labbé C. et al. A hydroponic-based bioassay to facilitate *Plasmodiophora brassicae* phenotyping. Plant Dis. 2024;108:131–8 37536345 10.1094/PDIS-05-23-0959-RE

[32] Ueno H, Matsumoto E, Aruga D. et al. Molecular characterization of the CRa gene conferring clubroot resistance in *Brassica rapa*. Plant Mol Biol. 2012;80:621–9 23054353 10.1007/s11103-012-9971-5

[33] Kato T, Hatakeyama K, Fukino N. et al. Fine mapping of the clubroot resistance gene CRb and development of a useful selectable marker in *Brassica rapa*. Breed Sci. 2013;63:116–24 23641188 10.1270/jsbbs.63.116 PMC3621437

[34] Hatakeyama K, et al. The tandem repeated organization of NB-LRR genes in the clubroot-resistant CRb locus in Brassica rapa L. Molecular genetics and genomics, 2017;292:397–405. 28013378 10.1007/s00438-016-1281-1

[35] Pang W, Fu P, Li X. et al. Identification and mapping of the clubroot resistance gene CRd in Chinese cabbage (*Brassica rapa* ssp. *pekinensis*). Front Plant Sci. 2018;9:653 29868100 10.3389/fpls.2018.00653 PMC5968122

[36] Wei X, Li J, Zhang X. et al. Fine mapping and functional analysis of major QTL, CRq for clubroot resistance in Chinese cabbage (*Brassica rapa* ssp. *pekinensis*). Agronomy. 2022;12:1172

[37] Chu M, Song T, Falk KC. et al. Fine mapping of Rcr1 and analyses of its effect on transcriptome patterns during infection by *Plasmodiophora brassicae*. BMC Genomics. 2014;15:1–20 25532522 10.1186/1471-2164-15-1166 PMC4326500

[38] Pang W, Zhang X, Ma Y. et al. Fine mapping and candidate gene analysis of CRA3. 7 conferring clubroot resistance in *Brassica rapa*. Theor Appl Genet. 2022;135:4541–8 36243892 10.1007/s00122-022-04237-2

[39] Sakamoto K., et al. Mapping of isolate-specific QTLs for clubroot resistance in Chinese cabbage (Brassica rapa L. ssp. pekinensis). Theor Appl Genet. 2008;117:759–67. 18612625 10.1007/s00122-008-0817-0

[40] Hirai M, Harada T, Kubo N. et al. A novel locus for clubroot resistance in *Brassica rapa* and its linkage markers. Theor Appl Genet. 2004;108:639–43 14551685 10.1007/s00122-003-1475-x

[41] Saito M. et al. Fine mapping of the clubroot resistance gene, Crr3, in Brassica rapa. Theor Appl Genet. 2006;114:81–91. 17039346 10.1007/s00122-006-0412-1

[42] Yu F, Zhang Y, Wang J. et al. Identification of two major QTLs in *Brassica napus* lines with introgressed clubroot resistance from turnip cultivar ECD01. Front Plant Sci. 2022;12:785989 35095960 10.3389/fpls.2021.785989 PMC8790046

[43] Hirani AH, Gao F, Liu J. et al. Combinations of independent dominant loci conferring clubroot resistance in all four turnip accessions (*Brassica rapa*) from the European clubroot differential set. Front Plant Sci. 2018;9:1628 30483286 10.3389/fpls.2018.01628 PMC6243934

[44] Fredua-Agyeman R. et al. QTL mapping and inheritance of clubroot resistance genes derived from *Brassica rapa* subsp. *rapifera* (ECD02) reveals resistance loci and distorted segregation ratios in two F (2) populations of different crosses. Front Plant Sci. 2020;11:899 32719696 10.3389/fpls.2020.00899 PMC7348664

[45] Chen J, Jing J, Zhan Z. et al. Identification of novel QTLs for isolate-specific partial resistance to *Plasmodiophora brassicae* in *Brassica rapa*. PLoS One. 2013;8:e85307 24376876 10.1371/journal.pone.0085307 PMC3869933

[46] Yu F. et al. Genotyping-by-sequencing reveals three QTL for clubroot resistance to six pathotypes of Plasmodiophora brassicae in Brassica rapa', Sci Rep. 2017;7:4516. 28674416 10.1038/s41598-017-04903-2 PMC5495781

[47] Huang Z. et al. Fine mapping of a clubroot resistance gene from turnip using SNP markers identified from bulked segregant RNA-Seq. Mol Breed. 2019;39:131. 10.3389/fpls.2017.01448 PMC5581393 28894454

[48] Kong L, Yang Y, Zhang Y. et al. Genetic mapping and characterization of the clubroot resistance gene BraPb8. 3 in *Brassica rapa*. Int J Mol Sci. 2024;25:10462 39408790 10.3390/ijms251910462 PMC11477069

[49] Laila R, et al. Mapping of a novel clubroot resistance QTL using ddRAD-seq in Chinese cabbage (Brassica rapa L.). BMC plant biology. 2019;19:1–9. 30621588 10.1186/s12870-018-1615-8 PMC6325862

[50] Karim MdM, et al. Two clubroot-resistance genes, Rcr3 and Rcr9 wa, mapped in Brassica rapa using bulk segregant RNA sequencing'. Int J Mol Sci. 2020;21:5033 32708772 10.3390/ijms21145033 PMC7404267

[51] Suwabe K, Tsukazaki H, Iketani H. et al. Identification of two loci for resistance to clubroot (*Plasmodiophora brassicae* Woronin) in *Brassica rapa* L. Theor Appl Genet. 2003;107:997–1002 12955203 10.1007/s00122-003-1309-x

[52] Yang S, Wang X, Wang Z. et al. A chromosome-level reference genome facilitates the discovery of clubroot resistant gene Crr5 in Chinese cabbage. Hortic Res. 2025;12:uhae338 40046320 10.1093/hr/uhae338 PMC11879649

[53] Suwabe K, Tsukazaki H, Iketani H. et al. Simple sequence repeat-based comparative genomics between *Brassica rapa* and *Arabidopsis* thaliana: the genetic origin of clubroot resistance. Genetics. 2006;173:309–19 16723420 10.1534/genetics.104.038968 PMC1461432

[54] Chang A, Lamara M, Wei Y. et al. Clubroot resistance gene Rcr6 in *Brassica nigra* resides in a genomic region homologous to chromosome A08 in B. Rapa. BMC Plant Biol. 2019;19:1–11 31142280 10.1186/s12870-019-1844-5 PMC6542104

[55] Dakouri A, Zhang X, Peng G. et al. Analysis of genome-wide variants through bulked segregant RNA sequencing reveals a major gene for resistance to *Plasmodiophora brassicae* in *Brassica oleracea*. Sci Rep. 2018;8:17657 30518770 10.1038/s41598-018-36187-5 PMC6281628

[56] Rocherieux J, Glory P, Giboulot A. et al. Isolate-specific and broad-spectrum QTLs are involved in the control of clubroot in *Brassica oleracea*. Theor Appl Genet. 2004;108:1555–63 15007504 10.1007/s00122-003-1580-x

[57] Nagaharu U . Genome analysis in *Brassica* with special reference to the experimental formation of B. Napus and peculiar mode of fertilization. Jpn J Bot. 1935;7:389–452

[58] Yang Z, Jiang Y, Gong J. et al. R gene triplication confers European fodder turnip with improved clubroot resistance. Plant Biotechnol J. 2022;20:1502–17 35445530 10.1111/pbi.13827 PMC9342621

[59] Karim MdM and Yu F. Identification of QTLs for resistance to 10 pathotypes of Plasmodiophora brassicae in Brassica oleracea cultivar ECD11 through genotyping-by-sequencing. Theor Appl Genet, 2023;136 :249. 37982891 10.1007/s00122-023-04483-y PMC10661809

[60] Voorrips RE, Jongerius MC, Kanne HJ. Mapping of two genes for resistance to clubroot (*Plasmodiophora brassicae*) in a population of doubled haploid lines of *Brassica oleracea* by means of RFLP and AFLP markers. Theor Appl Genet. 1997;94:75–82 19352748 10.1007/s001220050384

[61] Peng L, Zhou L, Li Q. et al. Identification of quantitative trait loci for clubroot resistance in *Brassica oleracea* with the use of *Brassica* SNP microarray. Front Plant Sci. 2018;9:822 29967632 10.3389/fpls.2018.00822 PMC6015909

[62] Nagaoka T, Doullah MAU, Matsumoto S. et al. Identification of QTLs that control clubroot resistance in *Brassica oleracea* and comparative analysis of clubroot resistance genes between B. Rapa and B. Oleracea. Theor Appl Genet. 2010;120:1335–46 20069415 10.1007/s00122-010-1259-z

[63] Moriguchi K. et al. A genetic map based on RAPD, RFLP, isozyme, morphological markers and QTL analysis for clubroot resistance in *Brassica oleracea*. Breed Sci. 1999;49:257–65

[64] Zhang H, Liu X, Zhou J. et al. Identification of clubroot (*Plasmodiophora brassicae*) resistance loci in Chinese cabbage (*Brassica rapa* ssp. *pekinensis*) with recessive character. Genes. 2024;15:274 38540333 10.3390/genes15030274 PMC10970103

[65] Piao Z, Ramchiary N, Lim YP. Genetics of clubroot resistance in *Brassica* species. J Plant Growth Regul. 2009;28:252–64

[66] Landry BS, Hubert N, Crete R. et al. A genetic map for *Brassica oleracea* based on RFLP markers detected with expressed DNA sequences and mapping of resistance genes to race 2 of *Plasmodiophora brassicae* (Woronin). Genome. 1992;35:409–20

[67] Laurens F, Thomas G. Inheritance of resistance to clubroot (*Plasmodiophora brassicae* Wor.) in kale (*Brassica oleracea* ssp. *acephala*). Hereditas. 1993;119:253–62

[68] Neik TX, Barbetti MJ, Batley J. Current status and challenges in identifying disease resistance genes in *Brassica napus*. Front Plant Sci. 2017;8:1788 29163558 10.3389/fpls.2017.01788 PMC5681527

[69] Manzanares-Dauleux MJ, Delourme R, Baron F. et al. Mapping of one major gene and of QTLs involved in resistance to clubroot in *Brassica napus*. Theor Appl Genet. 2000;101:885–91

[70] Wagner G, Laperche A, Lariagon C. et al. Resolution of quantitative resistance to clubroot into QTL-specific metabolic modules. J Exp Bot. 2019;70:5375–90 31145785 10.1093/jxb/erz265 PMC6793449

[71] Botero-Ramrez A. et al. Clubroot symptoms and resting spore production in a doubled haploid population of oilseed rape (*Brassica napus*) are controlled by four main QTLs. Front Plant Sci. 2020;11:604527 33391316 10.3389/fpls.2020.604527 PMC7773761

[72] Wang Z, Megha S, Kebede B. et al. Genetic and molecular analysis reveals that two major loci and their interaction confer clubroot resistance in canola introgressed from rutabaga. Plant Genome. 2022;15:e20241 35818693 10.1002/tpg2.20241 PMC12806973

[73] Hasan MJ, Rahman H. Genetics and molecular mapping of resistance to *Plasmodiophora brassicae* pathotypes 2, 3, 5, 6, and 8 in rutabaga (*Brassica napus* var. *napobrassica*). Genome. 2016;59:805–15 27549861 10.1139/gen-2016-0034

[74] Fredua-Agyeman R, Yu Z, Hwang S-F. et al. Genome-wide mapping of loci associated with resistance to clubroot in *Brassica napus* ssp. *napobrassica* (rutabaga) accessions from Nordic countries. Front Plant Sci. 2020;11:742 32595668 10.3389/fpls.2020.00742 PMC7303339

[75] Bhattacharya I, Dutta S, Mondal S. et al. Clubroot disease on *Brassica* crops in India. Can J Plant Pathol. 2014;36:154–60

[76] Hasan MJ, Rahman H. Resynthesis of *Brassica juncea* for resistance to *Plasmodiophora brassicae* pathotype 3. Breed Sci. 2018;68:385–91 30100807 10.1270/jsbbs.18010 PMC6081302

[77] Fredua-Agyeman R, Hwang SF, Strelkov SE. et al. Potential loss of clubroot resistance genes from donor parent *Brassica rapa* subsp. *rapifera* (ECD04) during doubled haploid production. Plant Pathol. 2018;67:892–901

[78] Diederichsen E, Sacristan MD. Disease response of resynthesized *Brassica napus* L. lines carrying different combinations of resistance to *Plasmodiophora brassicae* Wor. Plant Breed. 1996;115:5–10

[79] Hwang SF. et al. *Plasmodiophora brassicae*: a review of an emerging pathogen of the Canadian canola (*Brassica napus*) crop. Mol Plant Pathol. 2012;13:105–13 21726396 10.1111/j.1364-3703.2011.00729.x PMC6638701

[80] Gustafsson M, Falt A-S. Genetic studies on resistance to clubroot in *Brassica napus*. Ann Appl Biol. 1986;108:409–15

[81] Chalhoub B, Denoeud F, Liu S. et al. Early allopolyploid evolution in the post-Neolithic *Brassica napus* oilseed genome. Science. 2014;345:950–3 25146293 10.1126/science.1253435

[82] Belser C, Istace B, Denis E. et al. Chromosome-scale assemblies of plant genomes using nanopore long reads and optical maps. Nat Plants. 2018;4:879–87 30390080 10.1038/s41477-018-0289-4

[83] Sun D, Wang C, Zhang X. et al. Draft genome sequence of cauliflower (*Brassica oleracea* L. var. *botrytis*) provides new insights into the C genome in *Brassica* species. Hortic Res. 2019;6:82 31645943 10.1038/s41438-019-0164-0 PMC6804732

[84] Cai X, Chang L, Zhang T. et al. Impacts of allopolyploidization and structural variation on intraspecific diversification in *Brassica rapa*. Genome Biol. 2021;22:166 34059118 10.1186/s13059-021-02383-2 PMC8166115

[85] Amas JC. et al. Key advances in the new era of genomics-assisted disease resistance improvement of *Brassica* species. Phytopathology. 2023;113:771–85 36324059 10.1094/PHYTO-08-22-0289-FI

[86] Wang X. et al. The genome of the mesopolyploid crop species *Brassica rapa*. Nat Genet. 2011;43:1035–9 21873998 10.1038/ng.919

[87] Dangl JL, Jones JDG. Plant pathogens and integrated defence responses to infection. Nature. 2001;411:826–33 11459065 10.1038/35081161

[88] Ngou BP. et al. Mutual potentiation of plant immunity by cell-surface and intracellular receptors. Nature. 2021;592:110–5 33692545 10.1038/s41586-021-03315-7

[89] Greeff C, Roux M, Mundy J. et al. Receptor-like kinase complexes in plant innate immunity. Front Plant Sci. 2012;3:29126 10.3389/fpls.2012.00209 PMC3426755 22936944

[90] Grund E, Tremousaygue D, Deslandes L. Plant NLRs with integrated domains: unity makes strength. Plant Physiol. 2019;179:1227–35 30530739 10.1104/pp.18.01134 PMC6446777

[91] Dodds PN, Rathjen JP. Plant immunity: towards an integrated view of plant–pathogen interactions. Nat Rev Genet. 2010;11:539–48 20585331 10.1038/nrg2812

[92] Nelson R, Wiesner-Hanks T, Wisser R. et al. Navigating complexity to breed disease-resistant crops. Nat Rev Genet. 2018;19:21–33 29109524 10.1038/nrg.2017.82

[93] Li L, Luo Y, Chen B. et al. A genome-wide association study reveals new loci for resistance to clubroot disease in *Brassica napus*. Front Plant Sci. 2016;7:1483 27746804 10.3389/fpls.2016.01483 PMC5044777

[94] Zhang H, Ma X, Liu X. et al. Identification and fine-mapping of clubroot (*Plasmodiophora brassicae*) resistant QTL in *Brassica rapa*. Horticulturae. 2022;8:66

[95] Desta ZA, Ortiz R. Genomic selection: genome-wide prediction in plant improvement. Trends Plant Sci. 2014;19:592–601 24970707 10.1016/j.tplants.2014.05.006

[96] Cheng F, Mandáková T, Wu J. et al. Deciphering the diploid ancestral genome of the mesohexaploid *Brassica rapa*. Plant Cell. 2013;25:1541–54 23653472 10.1105/tpc.113.110486 PMC3694691

[97] Lee J, Izzah NK, Choi BS. et al. Genotyping-by-sequencing map permits identification of clubroot resistance QTLs and revision of the reference genome assembly in cabbage (*Brassica oleracea* L.). DNA Res. 2016;23:29–41 26622061 10.1093/dnares/dsv034 PMC4755525

[98] Hatakeyama K, Suwabe K, Tomita RN. et al. Identification and characterization of Crr1a, a gene for resistance to clubroot disease (*Plasmodiophora brassicae* Woronin) in *Brassica rapa* L. PLoS One. 2013;8:e54745 23382954 10.1371/journal.pone.0054745 PMC3559844

[99] Chen J, Li J, Ma M. et al. Improvement of resistance to clubroot disease in the Ogura CMS restorer line R2163 of *Brassica napus*. Plan Theory. 2022;11:2413 10.3390/plants11182413 PMC9504965 36145814

[100] Guo Y, Li B, Li M. et al. Efficient marker-assisted breeding for clubroot resistance in elite pol-CMS rapeseed varieties by updating the PbBa8.1 locus. Mol Breed. 2022;42:41 37313506 10.1007/s11032-022-01305-9 PMC10248692

[101] Zhan Z, Jiang Y, Shah N. et al. Association of clubroot resistance locus PbBa8. 1 with a linkage drag of high erucic acid content in the seed of the European turnip. Front Plant Sci. 2020;11:810 32595684 10.3389/fpls.2020.00810 PMC7301908

[102] Mehraj H, Akter A, Miyaji N. et al. Genetics of clubroot and *Fusarium* wilt disease resistance in *Brassica* vegetables: the application of marker assisted breeding for disease resistance. Plan Theory. 2020;9:726 10.3390/plants9060726 PMC7355935 32526827

[103] Aissiou F, Laperche A, Falentin C. et al. A novel *Brassica rapa* L. genetic diversity found in Algeria. Euphytica. 2018;214:241

[104] Ludwig-Mller J. et al. A novel methyltransferase from the intracellular pathogen *Plasmodiophora brassicae* methylates salicylic acid. Mol Plant Pathol. 2015;16:349–64 25135243 10.1111/mpp.12185 PMC6638400

[105] Lee S-S et al. Perspective Chapter: Creation and Evolution of Intergeneric Hybrids between Brassica Rapa and Raphanus Sativus. Brassica - Recent Advances. IntechOpen, 2023. Crossref.

[106] Crisp P, Crute IR, Sutherland RA. et al. The exploitation of genetic resources of *Brassica oleracea* in breeding for resistance to clubroot (*Plasmodiophora brassicae*). Euphytica. 1989;42:215–26

[107] Peng G, Falk KC, Gugel RK. et al. Sources of resistance to *Plasmodiophora brassicae* (clubroot) pathotypes virulent on canola. Can J Plant Pathol. 2014;36:89–99

[108] Hasan J. et al. Screening of *Brassica* germplasm for resistance to *Plasmodiophora brassicae* pathotypes prevalent in Canada for broadening diversity in clubroot resistance. Can J Plant Sci. 2012;92:501–15

[109] Xie Q, Wei X, Liu Y. et al. Germplasm enhancement and identification of loci conferring resistance against *Plasmodiophora brassicae* in broccoli. Genes. 2022;13:1600 36140766 10.3390/genes13091600 PMC9498593

[110] Ma Y, Wang H, Song J. et al. Identification of clubroot-resistant germplasm in a radish (*Raphanus sativus* L.) Core collection. Agronomy. 2024;14:157

[111] Cao T, Manolii VP, Strelkov SE. et al. Virulence and spread of *Plasmodiophora brassicae* [clubroot] in Alberta, Canada. Can J Plant Pathol. 2009;31:321–9

[112] Zhu M, Yang L, Zhang Y. et al. Introgression of clubroot resistant gene into *Brassica oleracea* L. from *Brassica rapa* based on homoeologous exchange. Hortic Res. 2022;9:uhac195 37180031 10.1093/hr/uhac195 PMC10167419

[113] Been CG, Park HG. Application of ovule culture to the production of intergeneric hybrids between Brassica and Raphanus. Korean J Hortic Sci. 1984;25:100–8

[114] Liu Y, Xu A, Liang F. et al. Screening of clubroot-resistant varieties and transfer of clubroot resistance genes to *Brassica napus* using distant hybridization. Breed Sci. 2018;68:258–67 29875610 10.1270/jsbbs.17125 PMC5982190

[115] Ren W, Li Z, Han F. et al. Utilization of Ogura CMS germplasm with the clubroot resistance gene by fertility restoration and cytoplasm replacement in *Brassica oleracea* L. Hortic Res. 2020;7:61 32377352 10.1038/s41438-020-0282-8 PMC7193625

[116] Primard-Brisset C, Poupard JP, Horvais R. et al. A new recombined double low restorer line for the Ogu-INRA cms in rapeseed (*Brassica napus* L.). Theor Appl Genet. 2005;111:736–46 15965648 10.1007/s00122-005-2059-8

[117] Ochoa JC, Mukhopadhyay S, Bieluszewski T. et al. Natural variation in *Arabidopsis* responses to *Plasmodiophora brassicae* reveals an essential role for resistance to Plasmodiophora brasssicae 1 (RPB1). Plant J. 2023;116:1421–40 37646674 10.1111/tpj.16438

[118] Zhou X, Zhong T, Wu M. et al. Multiomics analysis of a resistant European turnip ECD04 during clubroot infection reveals key hub genes underlying resistance mechanism. Front Plant Sci. 2024;15:1396602 38845850 10.3389/fpls.2024.1396602 PMC11153729

[119] Hu H, Zhang Y, Yu F. A CRISPR/Cas9-based vector system enables the fast breeding of selection-marker-free canola with Rcr1-rendered clubroot resistance. J Exp Bot. 2024;75:1347–63 37991105 10.1093/jxb/erad471 PMC10901203

[120] Hongge J, Omar AA, Orbović V. et al. Biallelic editing of the LOB1 promoter via CRISPR/Cas9 creates canker-resistant ‘Duncan’ grapefruit. Phytopathology. 2022;112:308–14 34213958 10.1094/PHYTO-04-21-0144-R

[121] Peng A, Chen S, Lei T. et al. Engineering canker-resistant plants through CRISPR/Cas9-targeted editing of the susceptibility gene Cs LOB 1 promoter in citrus. Plant Biotechnol J. 2017;15:1509–19 28371200 10.1111/pbi.12733 PMC5698050

[122] Zhou Q, Galindo-González L, Hwang S-F. et al. Application of genomics and transcriptomics to accelerate development of clubroot resistant canola. Can J Plant Pathol. 2021;43:189–208

[123] Quadrana L, Colot V. Plant transgenerational epigenetics. Annu Rev Genet. 2016;50:467–91 27732791 10.1146/annurev-genet-120215-035254

[124] Summanwar A, et al. 'Identification of lncRNAs responsive to infection by Plasmodiophora brassicae in clubroot-susceptible and-resistant Brassica napus lines carrying resistance introgressed from rutabaga', Mol Plant Microbe Interact, 2019;32:1360–77. 31090490 10.1094/MPMI-12-18-0341-R

[125] Zhu H. et al. Integrating long noncoding RNAs and mRNAs expression profiles of response to Plasmodiophora brassicae infection in Pakchoi (Brassica campestris ssp. chinensis Makino). Plos one. 2019;14:e0224927 31805057 10.1371/journal.pone.0224927 PMC6894877

[126] Liégard B. et al. 'Quantitative resistance to clubroot infection mediated by transgenerational epigenetic variation in Arabidopsis', New Phytol. 2019;222:468–79. 30393890 10.1111/nph.15579 PMC6587750

[127] Gravot A, et al. 'Two adjacent NLR genes conferring quantitative resistance to clubroot disease in Arabidopsis are regulated by a stably inherited epiallelic variation', Plant Commun. 2024;5 10.1016/j.xplc.2024.100824 PMC11121752 38268192

[128] Fitz-James MH, Cavalli G. Molecular mechanisms of transgenerational epigenetic inheritance. Nat Rev Genet. 2022;23:325–41 34983971 10.1038/s41576-021-00438-5 PMC7619059

[129] Johannes F, Porcher E, Teixeira FK. et al. Assessing the impact of transgenerational epigenetic variation on complex traits. PLoS Genet. 2009;5:e1000530 19557164 10.1371/journal.pgen.1000530 PMC2696037

[130] Watson IA and Singh D (1952), The future for rust resistant wheat in Australia.

[131] Baloch A, Shah N, Idrees F. et al. Pyramiding of triple clubroot resistance loci conferred superior resistance without negative effects on agronomic traits in *Brassica napus*. Physiol Plant. 2024;176:e14414 38956798 10.1111/ppl.14414

[132] Tomita H, Shimizu M, Asad-ud Doullah M. et al. Accumulation of quantitative trait loci conferring broad-spectrum clubroot resistance in *Brassica oleracea*. Mol Breed. 2013;32:889–900

[133] Wen R, Song T, Gossen BD. et al. Comparative transcriptome analysis of canola carrying a single vs stacked resistance genes against clubroot. Front Plant Sci. 2024;15:1358605 38835867 10.3389/fpls.2024.1358605 PMC11148231

[134] Wen R, Song T, Tonu NN. et al. Resilience of canola to *Plasmodiophora brassicae* (clubroot) Pathotype 3H under different resistance genes and initial inoculum levels. Plan Theory. 2024;13:1540 10.3390/plants13111540 PMC11174560 38891348

[135] Matsumoto E, Ueno H, Aruga D. et al. Accumulation of three clubroot resistance genes through marker-assisted selection in Chinese cabbage (*Brassica rapa* ssp. pekinensis). J Jpn Soc Hortic Sci. 2012;81:184–90

[136] Tonu NN, Wen R, Song T. et al. Canola with stacked genes shows moderate resistance and resilience against a field population of *Plasmodiophora brassicae* (clubroot) pathotype X. Plan Theory. 2023;12:726 10.3390/plants12040726 PMC9960129 36840074

[137] Song S, Hong J-E, Hossain MR. et al. Development of clubroot resistant cabbage line through introgressing six CR loci from Chinese cabbage via interspecific hybridization and embryo rescue. Sci Hortic. 2022;300:111036

[138] Shah N. et al. Genetic variation analysis of field isolates of clubroot and their responses to *Brassica napus* lines containing resistant genes CRb and PbBa8. 1 and their combination in homozygous and heterozygous state. Mol Breed. 2019;39:1–11

[139] Li X, Wei Y, Ma Y. et al. Marker-assisted pyramiding of CRa and CRd genes to improve the clubroot resistance of *Brassica rapa*. Genes. 2022;13:2414 36553679 10.3390/genes13122414 PMC9777773

[140] Wang W, Qin L, Zhang W. et al. WeiTsing, a pericycle-expressed ion channel, safeguards the stele to confer clubroot resistance. Cell. 2023;186:2656–2671.e18 37295403 10.1016/j.cell.2023.05.023

[141] Diederichsen E, Frauen M, Linders EGA. et al. Status and perspectives of clubroot resistance breeding in crucifer crops. J Plant Growth Regul. 2009;28:265–81

[142] Zhang C, du C, Li Y. et al. Advances in biological control and resistance genes of Brassicaceae clubroot disease-the study case of China. Int J Mol Sci. 2023;24:785 36614228 10.3390/ijms24010785 PMC9821010

[143] Strelkov SE, Hwang S-F, Manolii VP. et al. Emergence of new virulence phenotypes of *Plasmodiophora brassicae* on canola (*Brassica napus*) in Alberta, Canada. Eur J Plant Pathol. 2016;145:517–29

[144] Cao T, Manolii VP, Zhou Q. et al. Effect of canola (*Brassica napus*) cultivar rotation on *Plasmodiophora brassicae* pathotype composition. Can J Plant Sci. 2019;100:218–25

[145] Zamani-Noor N . Variation in pathotypes and virulence of *Plasmodiophora brassicae* populations in Germany. Plant Pathol. 2017;66:316–24

[146] Hwang SF. et al. Soil treatments and amendments for amelioration of clubroot of canola. Can J Plant Sci, 2011;91:999–1010.

[147] Peng G, Pageau D, Strelkov SE. et al. A> 2-year crop rotation reduces resting spores of *Plasmodiophora brassicae* in soil and the impact of clubroot on canola. Eur J Agron. 2015;70:78–84

[148] Hwang SF, Ahmed HU, Strelkov S. et al. Suppression of clubroot by dazomet fumigant. Can J Plant Sci. 2017;98:172–82

[149] Hennig BC, Hwang S-F, Manolii VP. et al. Evaluation of host resistance, hydrated lime, and weed control to manage clubroot in canola. Horticulturae. 2022;8:215

[150] Zhang J, Zhou X, Zhang Y. et al. Pre-soil fumigation with ammonium bicarbonate and lime modulates the rhizosphere microbiome to mitigate clubroot disease in Chinese cabbage. Front Microbiol. 2024;15:1376579 38686113 10.3389/fmicb.2024.1376579 PMC11057235

[151] Ahmed HU, Hwang SF, Strelkov SE. et al. Assessment of bait crops to reduce inoculum of clubroot (*Plasmodiophora brassicae*) of canola. Can J Plant Sci. 2011;91:545–51

[152] Hwang SF, Ahmed HU, Zhou Q. et al. Efficacy of V apam fumigant against clubroot (*Plasmodiophora brassicae*) of canola. Plant Pathol. 2014;63:1374–83

[153] Hwang SF, Ahmed HU, Strelkov SE. et al. Effects of rate and application method on the efficacy of metam sodium to reduce clubroot (*Plasmodiophora brassicae*) of canola. Eur J Plant Pathol. 2018;150:341–9

[154] Botero-Ramirez A, Kirk B, Strelkov SE. Optimizing clubroot management and the role of canola cultivar mixtures. Pathogens. 2024;13:640. 39204241 10.3390/pathogens13080640 PMC11357626

[155] Haun WJ, Hyten DL, Xu WW. et al. The composition and origins of genomic variation among individuals of the soybean reference cultivar Williams 82. Plant Physiol. 2011;155:645–55 21115807 10.1104/pp.110.166736 PMC3032456

[156] Swarup S, Cargill EJ, Crosby K. et al. Genetic diversity is indispensable for plant breeding to improve crops. Crop Sci. 2021;61:839–52

[157] Luo M, Xie L, Chakraborty S. et al. A five-transgene cassette confers broad-spectrum resistance to a fungal rust pathogen in wheat. Nat Biotechnol. 2021;39:561–6 33398152 10.1038/s41587-020-00770-x

[158] Ren Z, Li J, Zhang X. et al. Utilizing resequencing big data to facilitate *Brassica* vegetable breeding: tracing introgression pedigree and developing highly specific markers for clubroot resistance. Hortic Plant J. 2024;10:771–83

[159] Brim CA, Burton JW. Recurrent selection in soybeans. II. Selection for increased percent protein in seeds 1. Crop Sci. 1979;19:494–8

[160] Pandey S, Gardner C. Recurrent selection for population, variety, and hybrid improvement in tropical maize. Adv Agron. 1992;48:1–87

[161] Hollman KB, Hwang SF, Manolii VP. et al. Pathotypes of *Plasmodiophora brassicae* collected from clubroot resistant canola (*Brassica napus* L.) cultivars in western Canada in 2017-2018. Can J Plant Pathol. 2021;43:622–30

[162] Wu J, Prez-Lpez E. A multilayer strategy is needed to uncover the clubroot pathogen mysteries. Physiol Mol Plant Pathol. 2023;124:101971

[163] Ludwig-Mller J . What can we learn from -omics approaches to understand clubroot disease? Int J Mol Sci. 2022;23:6293 35682976 10.3390/ijms23116293 PMC9180986

[164] Fredua-Agyeman R, Hwang SF, Strelkov SE. et al. Identification of *Brassica* accessions resistant to ‘old’and ‘new’pathotypes of *Plasmodiophora brassicae* from Canada. Plant Pathol. 2019;68:708–18

[165] Zhang H, Feng J, Zhang S. et al. Resistance to *Plasmodiophora brassicae* in *Brassica rapa* and *Brassica juncea* genotypes from China. Plant Dis. 2015;99:776–9 30699533 10.1094/PDIS-08-14-0863-RE

[166] Zhang X, Han F, Li Z. et al. Map-based cloning and functional analysis of a major quantitative trait locus, BolC.Pb9.1, controlling clubroot resistance in a wild *Brassica* relative (*Brassica macrocarpa*). Theor Appl Genet. 2024;137:41 38305900 10.1007/s00122-024-04543-x

[167] Zhang Y. et al. Three preceding crops increased the yield of and inhibited clubroot disease in continuously monocropped Chinese cabbage by regulating the soil properties and rhizosphere microbial community. Microorganisms. 2022;10:799. 10.3390/microorganisms10040799 PMC9028536 35456849

[168] Zhang J. et al. Crop rotation with Marigold promotes soil bacterial structure to assist in mitigating clubroot incidence in Chinese cabbage. Plants (Basel). 2022;11:2295. 10.3390/plants11172295 PMC9460896 36079677

[169] Hwang SF, Cao T, Xiao Q. et al. Effects of fungicide, seeding date and seedling age on clubroot severity, seedling emergence and yield of canola. Can J Plant Sci. 2012;92:1175–86

[170] Jindo K, Goron TL, Pizarro-Tobías P. et al. Application of biostimulant products and biological control agents in sustainable viticulture: a review. Front Plant Sci. 2022;13:932311 36330258 10.3389/fpls.2022.932311 PMC9623300

[171] Liao J, Yuan Z, Wang X. et al. Magnesium oxide nanoparticles reduce clubroot by regulating plant defense response and rhizosphere microbial community of tumorous stem mustard (*Brassica juncea* var. *tumida*). Front Microbiol. 2024;15:1370427 38572228 10.3389/fmicb.2024.1370427 PMC10989686

[172] Struck C, Rsch S, Strehlow B. Control strategies of clubroot disease caused by *Plasmodiophora brassicae*. Microorganisms. 2022;10:620 35336194 10.3390/microorganisms10030620 PMC8949847

[173] Turganbay S, Aidarova S, Issayeva A. et al. Polyelectrolyte-surfactant mixture effects on bulk properties and antibacterial, cytotoxic activity of fine sulfur particles. Colloids Interfaces. 2024;8:65

[174] Hussain I, Zhao T, Wang Y. et al. Melatonin and copper oxide nanoparticles synergistically mitigate clubroot disease and enhance growth dynamics in *Brassica rapa*. Plant Physiol Biochem. 2024;215:109020 39128405 10.1016/j.plaphy.2024.109020

