# Supplementary material for: Clubroot resistant in cruciferous crops: recent advances in genes and QTLs identification and utilization
Source: Hortic Res. 2025 Apr 16;12(7):uhaf105. doi: 10.1093/hr/uhaf105 (PMC12096309; doi:10.1093/hr/uhaf105)
Supplement: Web_Material_uhaf105 [file web_material_uhaf105.docx]

**Supplementary Data Table S1.** The top 20 authors in clubroot ranked by publications, 1990-2024.

| **Rank** | **Author** | **Publications** | **Citations** | **g _index** |
| --- | --- | --- | --- | --- |
| 1 | STRELKOV SE | 109 | 3617 | 56 |
| 2 | HWANG SF | 90 | 2737 | 49 |
| 3 | GOSSEN BD | 72 | 2585 | 49 |
| 4 | LUDWIG-MÜLLER J | 48 | 2415 | 48 |
| 5 | PENG G | 46 | 1992 | 44 |
| 6 | HIRAI M | 12 | 1369 | 12 |
| 7 | MCDONALD MR | 43 | 1289 | 35 |
| 8 | MATSUMOTO S | 12 | 1227 | 12 |
| 9 | HATAKEYAMA K | 13 | 1132 | 13 |
| 10 | MANZANARES-DAULEUX MJ | 29 | 1078 | 29 |
| 11 | YU F | 30 | 1029 | 30 |
| 12 | SUWABE K | 7 | 927 | 7 |
| 13 | DIXON GR | 12 | 908 | 12 |
| 14 | SIEMENS J | 15 | 881 | 15 |
| 15 | CAO T | 16 | 867 | 16 |
| 16 | PIAO Z | 33 | 849 | 29 |
| 17 | MANOLII VP | 29 | 832 | 28 |
| 18 | HOWARD RJ | 12 | 690 | 12 |
| 19 | ZHANG Y | 46 | 686 | 25 |
| 20 | LAHLALI R | 11 | 686 | 11 |

**Notes: g-index**: The total citations of the top g papers must be at least g^2^, highlighting highly cited papers.

**Supplementary Data Table S2.** Top 10 cited articles in clubroot research, 1990-2024.

| **Rank** | **Article** | **Author** | **Journal** | **Citations** | **Citations per Year** |
| --- | --- | --- | --- | --- | --- |
| 1 | The Occurrence and Economic Impact of *Plasmodiophora brassicae* and Clubroot Disease | Dixon (Dixon 2009) | Journal of Plant Growth Regulation | 380 | 22.35 |
| 2 | Simple Sequence Repeat-Based Comparative Genomics Between Brassica rapa and *Arabidopsis thaliana*: The Genetic Origin of Clubroot Resistance | Suwabe et al. (Keita Suwabe et al. 2006) | Genetics | 275 | 13.75 |
| 3 | Transcriptome Analysis of *Arabidopsis* Clubroots Indicate a Key Role for Cytokinins in Disease Development | Suwabe et al. (Siemens et al. 2006) | Molecular Plant-Microbe Interactions® | 254 | 12.70 |
| 4 | Life Cycle of *Plasmodiophora brassicae* | Kageyama & Asano. (Kageyama and Asano 2009) | Journal of Plant Growth Regulation | 236 | 13.88 |
| 5 | Variation in Virulence of *Plasmodiophora brassicae* in Japan Tested with Clubroot-resistant Cultivars of Chinese Cabbage (*Brassica rapa* L. ssp. pekinensis) | Kuginuki et al. (Kuginuki et al. 1999) | European Journal of Plant Pathology | 225 | 8.33 |
| 6 | Prevalence of *Plasmodiophora brassicae* in a spring oilseed rape growing area in central Sweden and factors influencing soil infestation levels | Wallenhammar (Wallenhammar 1996) | Plant Pathology | 209 | 6.97 |
| 7 | Status and Perspectives of Clubroot Resistance Breeding in Crucifer Crops | Diederichsen et al. (Diederichsen et al. 2009) | Journal of Plant Growth Regulation | 204 | 12.00 |
| 8 | Characterization of *Plasmodiophora brassicae* populations from Alberta, Canada | Strelkov et al. (Strelkov et al. 2006) | Canadian Journal of Plant Pathology | 184 | 9.20 |
| 9 | Identification of two loci for resistance to clubroot (*Plasmodiophora brassicae* Woronin) in *Brassica rapa* L | Suwabe et al. (K. Suwabe et al. 2003) | Theoretical and Applied Genetics | 176 | 7.65 |
| 10 | Identification and Characterization of Crr1a, a Gene for Resistance to Clubroot Disease (*Plasmodiophora brassicae* Woronin) in *Brassica rapa* L. | Hatakeyama et al. (Hatakeyama et al. 2013) | PLOS ONE | 167 | 12.85 |

**References**

Diederichsen, Elke, et al. (2009), 'Status and Perspectives of Clubroot Resistance Breeding in Crucifer Crops', *Journal of Plant Growth Regulation,* 28 (3), 265-81.

Dixon, Geoffrey R (2009), 'The occurrence and economic impact of Plasmodiophora brassicae and clubroot disease', *Journal of Plant Growth Regulation,* 28 (3), 194-202.

Hatakeyama, Katsunori, et al. (2013), 'Identification and Characterization of Crr1a, a Gene for Resistance to Clubroot Disease (Plasmodiophora brassicae Woronin) in Brassica rapa L', *PLOS ONE,* 8 (1), e54745.

Kageyama, Koji and Asano, Takahiro (2009), 'Life Cycle of Plasmodiophora brassicae', *Journal of Plant Growth Regulation,* 28 (3), 203-11.

Kuginuki, Yasuhisa, Yoshikawa, Hiroaki, and Hirai, Masashi (1999), 'Variation in Virulence of Plasmodiophora brassicae in Japan Tested with Clubroot-resistant Cultivars of Chinese Cabbage (Brassica rapa L. ssp. pekinensis)', *European Journal of Plant Pathology,* 105 (4), 327-32.

Siemens, Johannes, et al. (2006), 'Transcriptome Analysis of Arabidopsis Clubroots Indicate a Key Role for Cytokinins in Disease Development', *Molecular Plant-Microbe Interactions®,* 19 (5), 480-94.

Strelkov, SE, Tewari, JP, and Smith-Degenhardt, E (2006), 'Characterization of Plasmodiophora brassicae populations from Alberta, Canada', *Canadian Journal of Plant Pathology,* 28 (3), 467-74.

Suwabe, K., et al. (2003), 'Identification of two loci for resistance to clubroot (Plasmodiophora brassicae Woronin) in Brassica rapa L', *Theoretical and Applied Genetics,* 107 (6), 997-1002.

Suwabe, Keita, et al. (2006), 'Simple Sequence Repeat-Based Comparative Genomics Between Brassica rapa and Arabidopsis thaliana: The Genetic Origin of Clubroot Resistance', *Genetics,* 173 (1), 309-19.

Wallenhammar, A. C. (1996), 'Prevalence of in a spring oilseed rape growing area in central Sweden and factors influencing soil infestation levels', *Plant Pathology,* 45 (4), 710-19.
